# Prognostic and Predictive Effects of Positive Lymph Node Number or Ratio in NSCLC

**DOI:** 10.1038/s41598-017-00619-5

**Published:** 2017-04-03

**Authors:** Nan Ding, ZhaoFei Pang, Xiangwei Zhang, Cuicui Huang, Yufan Yang, Qi Liu, Jiajun Du

**Affiliations:** 1 Institute of Oncology, Shandong Provincial Hospital Affiliated to Shandong University, Jinan, China; 2 Department of Thoracic Surgery, Shandong Provincial Hospital Affiliated to Shandong University, Jinan, China

## Abstract

In the eighth TNM staging system proposal for NSCLC recently, classification of N stage is based on anatomical position of positive lymph nodes. We aimed to expand the sample volume to identify the value of positive lymph node number or ratio in prognosis and predictive effect for postoperative radiation. Clinicopathological characters of 109026 NSCLC patients were collected from the SEER Database. Kaplan-Meier curves and cox regression methods were used for survival analysis. Compared with positive lymph node number equal to 0, 1–3 and >3 groups were independent prognostic factors (1–3: HR 2.856, p < 0.001; >3: HR 3.358, p < 0.001), so as the 0–50% and >50% positive lymph node ratio groups (0–50%: HR 2.124, p < 0.001; >50%: HR 3.358, p < 0.001). And in the groups of N2&positive lymph node number ≥4 and N2&positive lymph node ratio >50%, postoperative radiation related to positive prognosis of NSCLC patients. In conclusion, positive lymph node number or ratio was associated with survival as an independent indicator in NSCLC. They also had predictive effects for postoperative radiation, while N nodal stage not.

## Introduction

Lung cancer remains as the most common malignancy with high mortality worldwide^1^. Non-small cell lung cancer (NSCLC) accounts for approximately 85% of all cases^2^, and the 5-year survival rate is still low despite great advance has been made in diagnosis and treatment.

Up to now, a series of prognostic indicators have been identified, but lymph node status still remains the important one currently for NSCLC patients^3^. Although the number of metastatic lymph nodes (MLNs) has been included in N staging system for breast, gastric and colorectal cancer. It has been reported number of positive lymph nodes had a better prognostic effect than anatomical position in these cancers^4^. Whereafter, due to the limited number of examined lymph node, the ratio of metastatic lymph nodes to total examined number was explored, and confirmed to have prognostic value for various tumors, even better than MLNs number^5–10^. The prognostic effects of positive lymph number and ratio for NSCLC have been reported in some studies^7, 11–13^, and here, we used the Surveillance, Epidemiology, and End Results (SEER) Database aiming to expand the sample volume and confirm their values in prognosis. Besides, we also searched their effects for patients at N1, N2 stage to clarify if they could be influenced by N stage.

Surgery is an effective way for NSCLC patients at early stage. However, among these operable patients, there are some harboring microscopic disease, and postoperative radiation might be performed aiming to reduce local recurrence and improve the outcome. Obviously, not all these patients could get benefits from it, some even could obtain detrimental results, so which group people should receive postoperative radiation was an important issue that have not been figured out. It is crucial to find predictive factors to determine the conduction of radiation after surgery. In 2006, Brian E. Lally and his colleagues searched SEER database and found postoperative radiation was related to an increase in survival in patients at N2 nodal stage but not in patients at N1 and N0 nodal stage^14^. As previously mentioned, positive lymph node number and ratio might have prognostic effects like N nodal stage, so we used a larger sample volume from 1988 to 2013 to further identify whether they have predictive values for postoperative radiation.

## Material and Methods

### Patients selection

The information about patients was collected from the Surveillance, Epidemiology, and End Results (SEER) database, a population-based cancer surveillance program covering approximately 28% of the population of the United States. Patients would be included if they met the following criteria: (1) patients >20 years old; (2) diagnosed with NSCLC pathologically; (3) receiving tumor resection only or radiation after surgery; (4) survival month more than 4 months. The criteria of patients exclusion was as followed: (1) at M1 stage; (2) without complete information about N stage; (3) at N3 stage; (4) without definitive number of examined and positive lymph node; (5) with controversial information (e.g patients at N1 or N2 stages with 0 positive lymph node).

### Clinical and follow-up data collection

In the process of selection, we also collected clinicopathological characteristics and follow-up information about patients, including age, gender, race, marital status, histological subtype, tumor size, N stage, differential degree, cancer position, treatment, year of diagnosis, number of examined and positive lymph node, survival status and survival months.

### Statistical analysis

For the optimal cutoff of positive lymph node number and ratio, we used χ2 scores which were calculated using the Cox proportional hazards regression model. We performed Kaplan-Meier (K-M) analysis to test if positive lymph node number and ratio were significant for prognosis or prediction for postoperative radiation in all patients and patients at different N stages. Hazard ratio with its 95% confidence interval for describing association of variables and survival was calculated by univariate and multivariate cox regression methods. All statistical calculations were performed by SPSS (version 20.0) software (Inc., Chicago, IL, USA), and a two-sided p ≤ 0.05 was considered to be significant.

### Ethical approval

The study was approved by ethic community of Shandong Provincial Hospital afflicted to Shandong University. All the experiments described here were performed in accordance with the approved guidelines.

## Results

### Characteristics of patients

**Figure 1:**
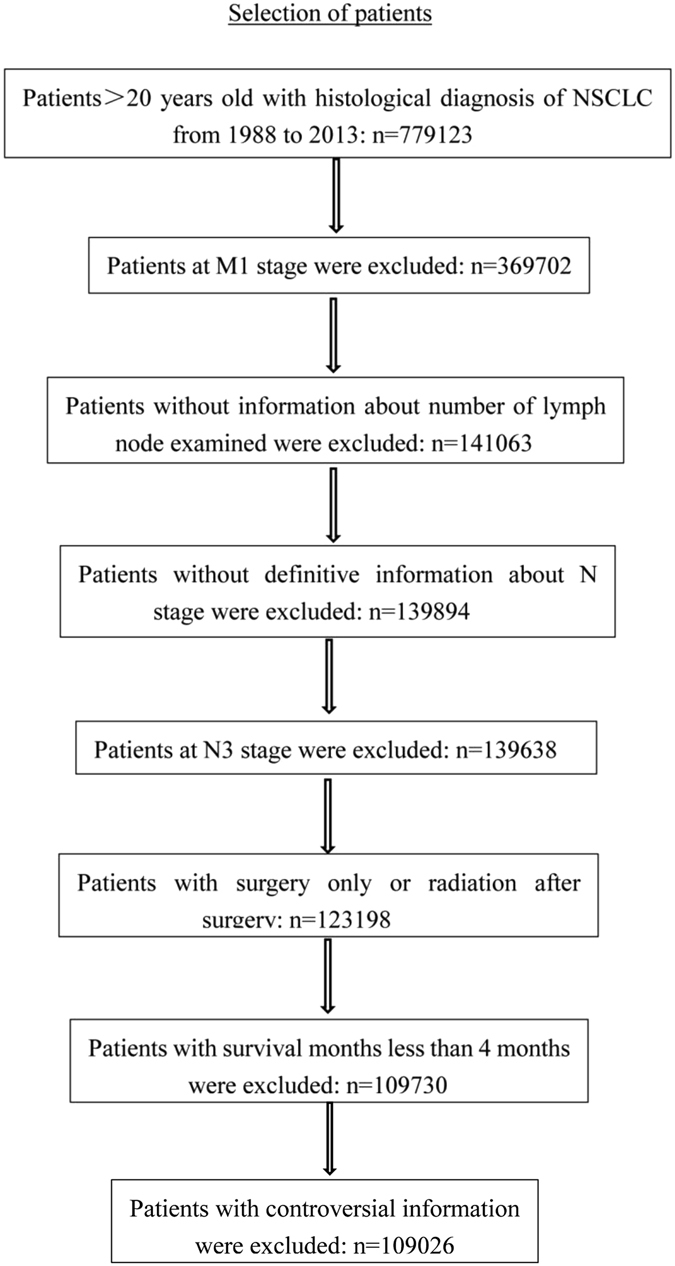
Flow chart of process of patients selection according to some inclusion and exclusion criteria. NSCLC: non-small cell lung cancer.

As presented in the flow chart of patients selection (Fig. 1), 109026 ones were included in our analysis finally according to the inclusion and exclusion criteria. Among them, there were 54651 (50.1%) women and 54375 (49.9%) men. And there were 40638 (37.3%) patients ≤65 years old and 68388 (62.7%) patients >65 years old. The survival time of these patients ranged from 5 to 311 months with the median of 44.5 months. Other detailed information about race, marital status, histological subtype, tumor size, N stage, differential degree, cancer position, treatment, year of diagnosis, number of examined and positive lymph node, survival status were listed in Table 1.

**Table 1:** Clinicopathological characteristics of all included NSCLC patients.

Characteristics	Number of patients	Characteristics	Number of patients
**All patients**	109026(100%)	**Differential degree**	
**Age**		Well	14450(13.3%)
≤65	40638(37.3%)	Moderately	42013(38.5%)
>65	68388(62.7%)	Poorly	38829(35.6%)
**Gender**		Undifferentiated	3616(3.3%)
Female	54651(50.1%)	unknown	10118(9.3%)
Male	54375(49.9%)	**Position of cancer**	
**Histological subtype**		left	47128(43.2%)
Adenocarcinoma	60751(55.7%)	right	61854(56.7%)
Squamous carcinoma	29335(26.9%)	other	44(<0.1%)
Others	18940(17.4%)	**Marital status**	
**Race**		married	65514(60.1%)
White	93249(85.5%)	Single,divorced,widowed	40376(37.0%)
Black	9098(8.3%)	unknown	3136(2.9%)
other	6518(6.0%)	**Survival status**	
unknown	161(0.1%)	Alive	48156(44.2%)
**Tumor size**		dead	60870(55.8%)
T ≤ 3 cm	65272(59.9%)	**Treatment**	
3 < T ≤ 5 cm	24236(22.2%)	Surgery only	95456(87.6%)
5 < T ≤ 7 cm	9332(8.6%)	Radiation after surgery	13570(12.4%)
T > 7 cm	4652(4.3%)	**Year of diagnosis**	
unknown	5534(5.1%)	1988–2003	46150(42.3%)
**N stage**		2004–2010	45059(41.3%)
N0	82836(76.0%)	2011–2013	17817(16.3%)
N1	15812(14.5%)	**Number of positive lymph node**	
N2	10378(9.5%)	0	82836(76.0%)
**Surgery type**		1–3	20407(18.7%)
Pneumonectomy	5294(4.9%)	≥4	5783(5.3%)
Lobectomy	79595(73.0%)	**Ratio of positive lymph node**	
Local resection	10567(9.7%)	0	82836(76.0%)
unknown	13570(12.4%)	0–50%	20941(19.2%)
		>50%	5249(4.8%)

### Cutoff values for positive lymph node number and ratio

We used Cox proportional hazards regression model to determine the cutoff values for positive lymph node number and ratio. In accordance with the maximalχ^2^ scores shown in Table 2, three nodes and 50% were chosen as optimal cutoff values for positive lymph node number and ratio respectively.

**Table 2:** Analysis of the number of positive lymph node and ratio of positive lymph node using the cox proportional hazards regression model.

	Cutoff value	x2 score	p value
Cutoff value for positive lymph node number	0, 1, ≥2	6547.625	<0.001
	0, 1–2, ≥3	6678.684	<0.001
	0, 1–3, ≥4	**6772.235**	<0.001
	0, 1–4, ≥5	6745.445	<0.001
	0, 1–5, ≥6	6697.326	<0.001
	0, 1–6, ≥7	6588.328	<0.001
Cutoff value for positive lymph node ratio(%)	0, 0–5, >5	6207.494	<0.001
	0, 0–10, >10	6547.309	<0.001
	0, 0–15, >15	6872.716	<0.001
	0, 0–20, >20	7143.444	<0.001
	0, 0–25, >25	7292.579	<0.001
	0, 0–30, >30	7343.819	<0.001
	0, 0–35, >35	7487.844	<0.001
	0, 0–40, >40	7425.814	<0.001
	0, 0–45, >45	7448.154	<0.001
	0, 0–50, >50	**7532.915**	<0.001
	0, 0–55, >55	7505.264	<0.001
	0, 0–60, >60	7366.118	<0.001

### Survival analysis of positive lymph node number and ratio

**Figure 2:**
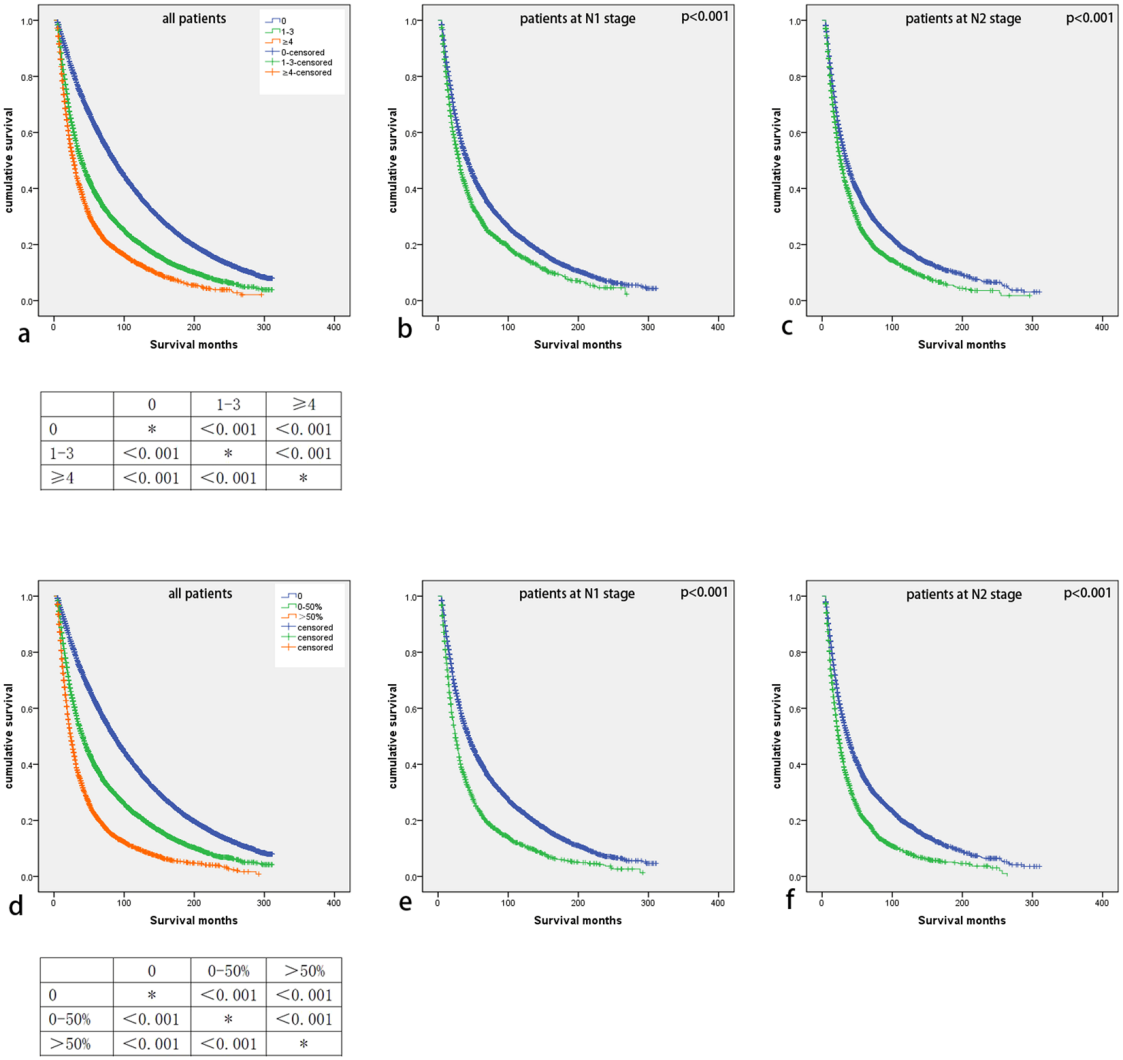
(**a**) Kaplan–Meier survival curve of positive lymph node number for all included patients and relevant log-rank analysis results; (**b**) Kaplan–Meier survival curve of positive lymph node number for patients at N1 stage; (**c**) Kaplan–Meier survival curve of positive lymph node number for patients at N2 stage; (**d**) Kaplan–Meier survival curve of positive lymph node ratio for all included patients and their log-rank analysis results; (**e**) Kaplan–Meier survival curve of positive lymph node ratio for patients at N1 stage; (**f**) Kaplan–Meier survival curve of positive lymph node ratio for patients at N2 stage.

When we analyzed the prognostic effects of positive lymph node number and ratio in all included NSCLC patients by K-M curves, we found they were both associated with overall survival (OS) (p < 0.001 for both) significantly (Fig. 2). Compared with positive lymph node number equal to 0, 1–3 and >3 positive lymph node groups were independent prognostic factors for NSCLC patients(1–3: HR 2.856, 95% CI 2.734–2.984, p < 0.001; >3: HR 3.358, 95% CI 3.224–3.499, p < 0.001), so as the 0–50% and >50% positive lymph node ratio groups (0–50%: HR 2.124, 95% CI 2.037–2.215, p < 0.001; >50%: HR 3.358, 95% CI 3.224–3.499, p < 0.001). And N stage was also an independent indicator for prognosis (positive lymph node number included: HR 2.834, 95% CI 2.709–2.966, p < 0.001; positive lymph node ratio included: HR 3.358, 95% CI 3.224–3.499, p < 0.001).

When stratified by N stage, positive lymph node number and ratio also related to prognosis of NSCLC patients at N1 stage or N2 stage (Fig. 2).

### Univariate and multivariate analysis of prognostic factors

After univariate analysis in searching significant factors for prognosis, a series indicators met the criteria and were included in multivariate analysis, such as age, gender, histological subtype, tumor size, N stage, differential degree, positive lymph node number and ratio. As presented in Table 3, these were all confirmed to be independent prognostic indicators for NSCLC patients except “other histological types” group.

**Table 3:** Univariate and multivariate analysis of prognostic factors for OS of NSCLC patients.

Characteristics	Univariate analysis		Multivariate analysis	
	HR (95%CI)	p	HR (95%CI)	p
**Age**				
≤65	1		1	
>65	1.608(1.580–1.636)	<0.001	1.650(1.619–1.682)	<0.001
**Gender**				
Female	1		1	
Male	1.381(1.359–1.403)	<0.001	1.248(1.226–1.271)	<0.001
**Histological subtype**				
Adenocarcinoma	1		1	
Squamous carcinoma	1.338(1.315–1.363)	<0.001	1.089(1.067–1.111)	<0.001
Others	0.984(0.962–1.006)	0.161	1.007(0.980–1.036)	0.611
**Tumor size**				
T ≤ 3 cm	1		1	
3 < T ≤ 5 cm	1.382(1.355–1.409)	<0.001	1.182(1.158–1.207)	<0.001
5 < T ≤ 7 cm	1.624(1.580–1.669)	<0.001	1.326(1.288–1.365)	<0.001
T > 7 cm	2.100(2.026–2.176)	<0.001	1.701(1.638–1.767)	<0.001
**N stage**				
N0	1		1	
N1	1.822(1.784–1.860)	<0.001	2.834(2.709–2.966)	<0.001
N2	2.249(2.194–2.305)	<0.001	3.358(3.224–3.499)	<0.001
**Differential degree**				
Well	1		1	
Moderately	1.556(1.510–1.602)	<0.001	1.383(1.341–1.427)	<0.001
Poorly	1.898(1.843–1.954)	<0.001	1.559(1.511–1.609)	<0.001
Undifferentiated	1.950(1.861–2.042)	<0.001	1.613(1.531–1.699)	<0.001
**Number of positive lymph node**				
0	1		1	
1–3	1.839(1.804–1.875)	<0.001	2.856(2.734–2.984)	<0.001
≥4	2.589(2.509–2.671)	<0.001	3.358(3.224–3.499)	<0.001
**Ratio of positive lymph node**				
0	1		1	
0–50%	1.792(1.758–1.826)	<0.001	2.124(2.037–2.215)	<0.001
>50%	2.956(2.865–3.050)	<0.001	3.358(3.224–3.499)	<0.001

### Predictive effect of positive lymph node number and ratio for postoperative radiation

**Figure 3:**
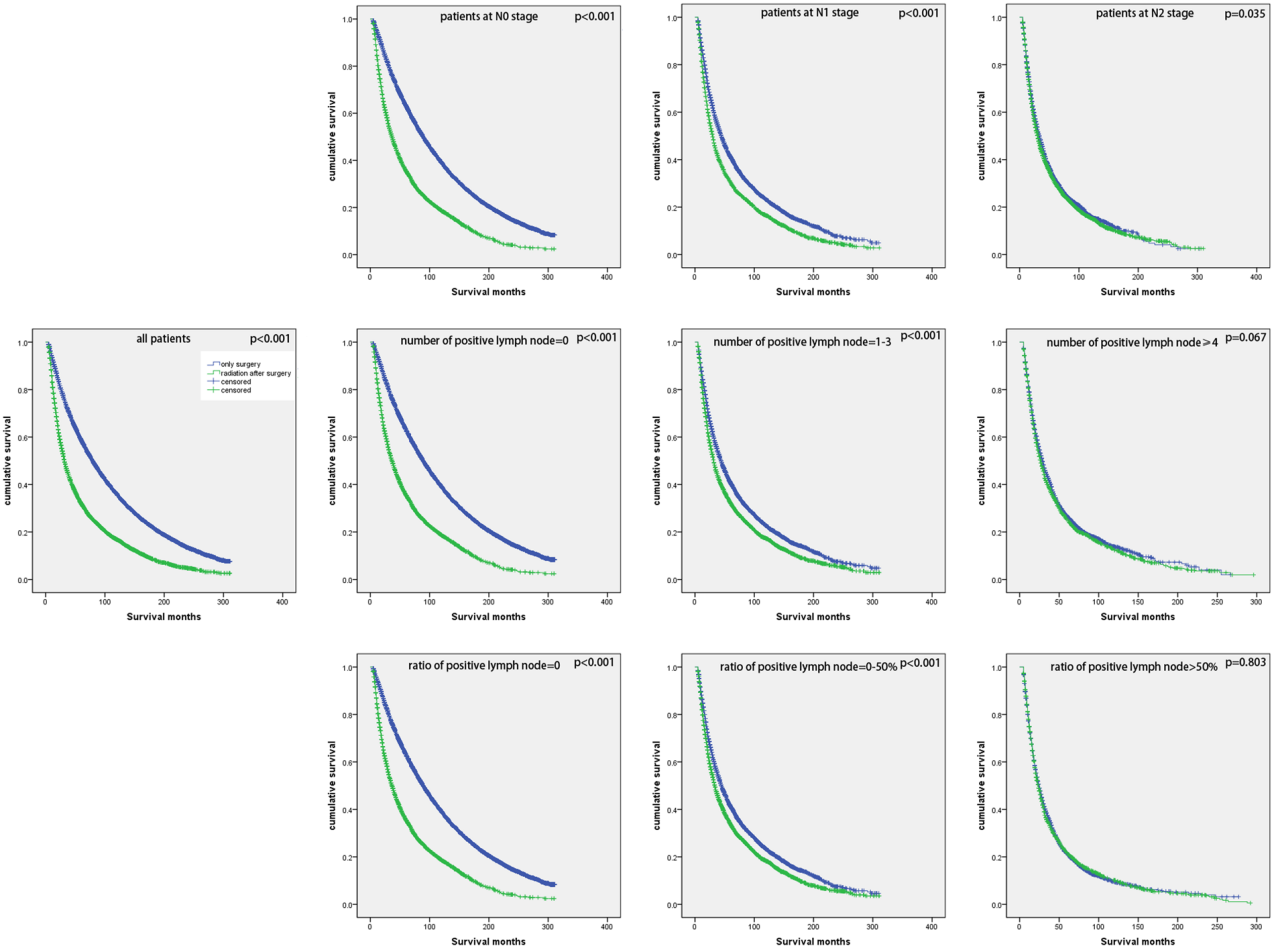
(**a**) Kaplan–Meier survival curve of postoperative radiation for all included patients; (**b–d**) Kaplan–Meier survival curves of postoperative radiation for patients at N0 stage, patients at N1 stage, patients at N2 stage; (**e–g**) Kaplan–Meier survival curves of postoperative radiation for patients with positive lymph node number = 0, patients with positive lymph node number = 1–3, patients with positive lymph node number ≥4; (**h–j**) Kaplan–Meier survival curves of postoperative radiation for patients with positive lymph node ratio = 0, patients with positive lymph node ratio = 0–50%, patients with positive lymph node ratio >50%.

In analysis for all included patients, we could see that postoperative radiation was correlated to poor survival compared with surgery without radiation (p < 0.001). The same results were seen in N0, N1, N2, positive number = 0, positive number = 1–3, positive ratio = 0 and positive ratio = 0–50% groups (p < 0.001 for all except N2 (p = 0.035)). But the results were not significant in positive number ≥4 and positive ratio >50% groups (p = 0.067, p = 0.803 respectively) (Fig. 3).

**Figure 4:**
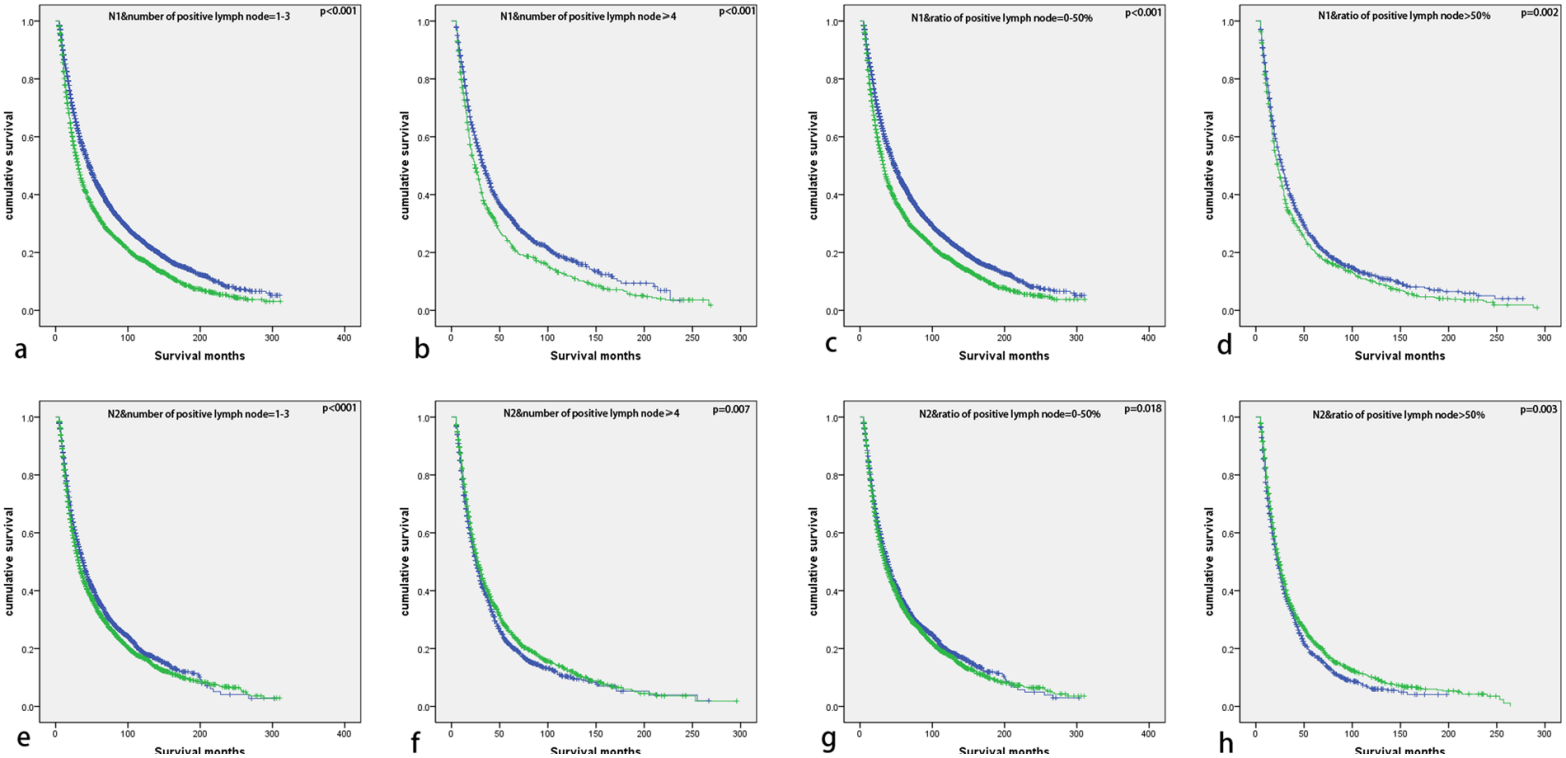
(**a**) Kaplan–Meier survival curve of postoperative radiation for patients with N1&positive lymph node number = 1–3; (**b**) Kaplan–Meier survival curve of postoperative radiation for patients with N1&positive lymph node number ≥4; (**c**) Kaplan–Meier survival curve of postoperative radiation for patients with N1&positive lymph node ratio = 0–50%; (**d**) Kaplan–Meier survival curve of postoperative radiation for patients with N1&positive lymph node ratio >50%; (**e**) Kaplan–Meier survival curve of postoperative radiation for patients with N2&positive lymph node number = 1–3; (**f**) Kaplan–Meier survival curve of postoperative radiation for patients with N2&positive lymph node number ≥4; (**g**) Kaplan–Meier survival curve of postoperative radiation for patients with N2&positive lymph node ratio = 0–50%; (**h**) Kaplan–Meier survival curve of postoperative radiation for patients with N2&positive lymph node ratio >50%.

As seen in Fig. 4, in the further subgroup analysis, we found in the groups of N2&positive lymph node number ≥4 and N2&positive lymph node ratio >50%, postoperative radiation related to positive prognosis of NSCLC patients (p = 0.007, p = 0.003 respectively). And in other subgroups, the results were opposite, but same to the result in analysis of all patients.

In order to validate the conclusion, we performed multivariate analysis including age, gender, histological subtype, tumor size, differential degree and treatment at patients with N2&positive lymph node number ≥4 or N2&positive lymph node ratio >50% (seen in Table 4). And the prognosis of patients receiving radiation after surgery was significantly better than the ones only undergoing surgery in the two groups (N2&positive lymph node number ≥4: HR 0.904, 95% CI 0.833–0.982, p = 0.016; N2&positive lymph node ratio > 50%: HR 0.869, 95% CI 0.795–0.949, p = 0.002).

**Table 4:** Multivariate analysis of treatment for patients with N2&positive lymph node number ≥4 or N2&positive lymph node ratio >50%.

Characteristics	multivariate analysis for N2&positive lymph node number ≥4		multivariate analysis for N2&positive lymph node ratio >50%	
	HR (95%CI)	p	HR (95%CI)	p
**Age**				
≤65	1		1	
>65	1.436(1.321–1.562)	<0.001	1.338(1.223–1.463)	<0.001
**Gender**				
Female	1		1	
Male	1.220(1.123–1.326)	<0.001	1.208(1.105–1.320)	<0.001
**Histological subtype**				
Adenocarcinoma	1		1	
Squamous carcinoma	0.919(0.824–1.024)	0.124	0.962(0.851–1.087)	0.535
Others	0.941(0.834–1.061)	0.320	0.879(0.771–1.003)	0.056
**Tumor size**				
T ≤ 3 cm	1		1	
3 < T ≤ 5 cm	1.162(1.355–1.409)	<0.001	1.172(1.060–1.296)	0.002
5 < T ≤ 7 cm	1.624(1.580–1.669)	<0.001	1.377(1.204–1.574)	<0.001
T > 7 cm	2.100(2.026–2.176)	<0.001	1.629(1.353–1.963)	<0.001
**Differential degree**				
Well	1		1	
Moderately	1.556(1.056–1.280)	0.002	1.303(1.056–1.608)	0.014
Poorly	1.213(1.078–1.365)	0.001	1.480(1.202–1.822)	<0.001
Undifferentiated	1.578(1.350–1.844)	<0.001	1.518(1.136–2.027)	0.005
**Treatment**				
Only surgery	1		1	
Radiation after surgery	0.904(0.833–0.982)	0.016	0.869(0.795–0.949)	0.002

## Discussion

In 2006, Fukui T and his colleagues firstly proved that the number of positive lymph node had significance in resected NSCLC patients^12^, and then some studies were conducted confirming the result^11, 13^. Soon after, researchers realized the number of examined lymph node would limit the maximal number of MLN, and sufficient lymph nodes should be sampled to analyze the lymph node status to guarantee the reliability. Accordingly, ratio of MLNs, the number of MLNs by the examined lymph node number, was explored and confirmed to have prognostic value in lung cancer in some studies^7, 15–17^. However, in the eighth TNM staging system proposal published recently, the determination of N stage is still based on anatomical position. So we searched SEER database covering approximately 28% of the population of the United States according to certain criteria, and 109026 patients were included finally. Through K-M curves and cox regression analysis methods, we got the result that positive lymph node number or ratio was associated with survival for NSCLC patients as an independent indicator. Even in the subgroup stratified by different N stage, the results were still significant. These all demonstrated that positive lymph node number or ratio are stronger prognostic parameters for patients with NSCLC. It would be better to consider the number or ratio of positive lymph node not only the anatomical position in the new TNM staging system.

Sometimes, postoperative radiation was performed for resected NSCLC patients to reduce local recurrence and improve survival. But results from a series of studies suggested that not all these patients would get benefits from radiation after surgery, some even would obtain detrimental result. In 1998, an article of meta-analysis containing nine studies with 2128 patients reported that whether postoperative had detrimental effect was determined by N nodal status. Significantly reduced survival was seen in patients at N0 and N1 stage, and patients at N2 stage seemed to advocate postoperative radiation but the result was not significant^18^. An updated meta-analysis with 10 studies published in 2005 had the same result^19^. Then in 2006, an authoritative article with 7465 NSCLC patients from SEER database demonstrated that postoperative radiation was associated with an increase in survival in patients at N2 stage significantly but not in patients at N0 or N1 stage^14^. As mentioned in the above paragraph, positive lymph node number or ratio might have better prognostic effect than N nodal stage, so we hypothesized that they perhaps had better predictive effect for postoperative radiation, either. Interestingly, when we expanded the sample volume, we obtained a different result as previously reported, postoperative radiation had detrimental effect for patients with any N nodal disease significantly. However, in the subgroups of positive lymph node number ≥4 and positive lymph node ratio >50%, the results were not significant. For further exploration, we divided these patients into 8 subgroups according to N stage and positive lymph node number or ratio: (1) N1&positive lymph node number =1–3; (2) N1&positive lymph node number ≥4; (3) N2&positive lymph node number = 1–3; (4) N2&positive lymph node number ≥4; (5) N1&positive lymph node ratio = 0–50%; (6) N1&positive lymph node ratio > 50%; (7) N2&positive lymph node ratio = 0–50%; (8) N2&positive lymph node ratio > 50%. And found that postoperative radiation would benefit patients in the subgroups of “N2&positive lymph node number ≥4” and “N2&positive lymph node ratio >50%”, patients in other ten groups obtained opposite results significantly. These suggested that the combination of N nodal stage and positive lymph node number or ratio was a good means to select potential NSCLC patients who could get benefit from postoperative radiation.

Although we had a large enough sample of NSCLC patients to conduct our analysis, there were some bias difficult to avoid due to the nature of respective study. And the SEER data is available data with heterogeneity and other limitations, we could not control the baseline of different groups to be the same. That is to say that those who used radiation might be quite different at the baseline from those without using radiation even among the NSCLC patients at the same N stage. So further exploration especially randomized clinical trials should be performed to confirm the result.

In conclusion, our study analyzed 109026 patients with NSCLC between 1988 and 2013 from SEER database, and got the result that positive lymph node number or ratio was associated with survival as an independent indicator in NSCLC. And they also had predictive effects for postoperative radiation. For NSCLC patients in subgroups of “N2&positive lymph node number ≥4” and “N2&positive lymph node ratio >50%”, postoperative radiation use had a positive effect on survival significantly. And we could not get the result only according to the N nodal stage.
